# Case Report: A Novel Deletion in the 11p15 Region Causing a Familial Beckwith–Wiedemann Syndrome

**DOI:** 10.3389/fgene.2021.621096

**Published:** 2021-02-19

**Authors:** Juan Chen, Jian Xu, Yang Yu, Ling Sun

**Affiliations:** Department of Assisted Reproductive Technology, Guangzhou Women and Children's Medical Center, Guangzhou Medical University, Guangzhou, China

**Keywords:** Beckwith–Wiedemann syndrome, 11p15, MLPA, azoospermia, imprinting

## Abstract

Beckwith–Wiedemann syndrome (BWS; OMIM 130650) is a human overgrowth and cancer susceptibility disorder with a wide clinical spectrum, which cannot be predicted based on genomic variants alone. Most reports on BWS cases focus on childhood patients. Studies on adult BWS patients are scarce. Our study reports a BWS family in which the disorder appears to be caused by deletion of *H19* and its upstream regulatory elements. Genetic analysis showed a heterozygous microdeletion (~chr11:2009895-2070570 (GRCh37)) in the patients. Maternal deletion in *H19* can result in loss of function of the IGF2-H19 imprinting control element, which leads to BWS. The male proband in this family was affected by the testicular anomaly and cryptorchidism. Early orchidopexy did not rescue his azoospermia, which might be not the consequence of cryptorchidism, but due to genetic defects associated with H19 deletion. In summary, our study gives some insights on the presentation of BWS in adulthood.

## Introduction

Beckwith–Wiedemann syndrome (BWS; OMIM 130650) is a human overgrowth disorder, characterized by macrosomia, hemihyperplasia, abdominal wall defects, macroglossia, and neonatal hypoglycemia (Choufani et al., 2010). Cryptorchidism is a common symptom in male BWS patients and adult male patients usually face subfertility problems (Cohen, 1971; Kosseff et al., 1976; Taylor, 1981; Watanabe and Yamanaka, 1990; Elliott et al., 1994; Gazzin et al., 2019).

**Figure 1:**
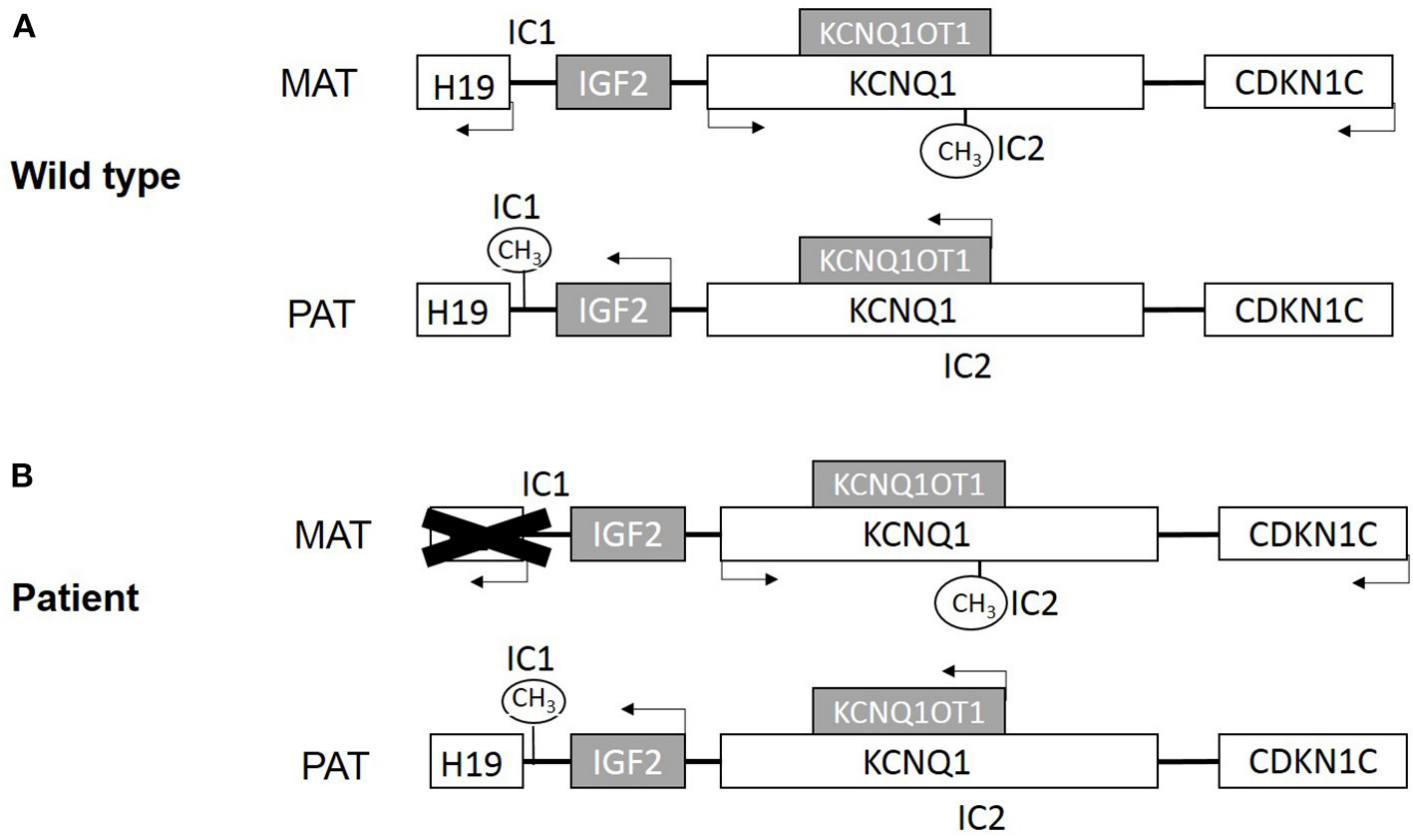
**(A)** Scheme of imprinted gene cluster in wild type. **(B)** Scheme of imprinted gene cluster in patient. MAT, maternal derived; PAT, paternal derived. White boxes present maternal expression genes, and gray present paternal expression genes. IC1 is paternal methylation and IC2 is maternal methylation.

BWS is associated with genetic and epigenetic changes on the chromosome 11p15 region (Choufani et al., 2010). There are two imprinting centers in the 11p15 region (Figure 1A). One includes a region that encodes a long noncoding RNA (lncRNA) *H19* and insulin-like growth factor 2 (*IGF2*) and is controlled by *H19/IGF2* intergenic differentially methylated region (*H19/IGF2*: IG DMR), which is also called imprinting control region 1 (IC1). Another includes *KCNQ1*, the regulatory lncRNA *KCNQ1OT1*, and *CDKN1C*. This region is controlled by the *KCNQ1OT1* transcription start site differentially methylated region (*KCNQ1OT1*: TSS DMR), which is called imprinting control region 2 (IC2) (Hark et al., 2000; Diaz-Meyer et al., 2003; Pandey et al., 2008).

Approximately 80% of BWS patients have a molecular defect in the 11p15 region, mostly due to abnormal DNA methylation (Choufani et al., 2010). Only 15% of these cases are inherited, and nearly half of them are associated with *CDKN1C* mutations (Algar et al., 2000; Li et al., 2001; Brioude et al., 2015).

Beyond that, chromosomal duplications, deletions, and translocations of the 11p15 region contribute to about 1% of BWS cases (Niemitz et al., 2004; Sparago et al., 2004; Prawitt et al., 2005b; Krzyzewska et al., 2019). Here, we report a BWS family with an uncommon DNA aberration, which results in deletion of *H19* and its upstream regulatory genes (Figure 1B). The male offspring underwent bilateral orchidopexy in childhood but still developed azoospermia.

## Case Presentation

**Figure 2:**
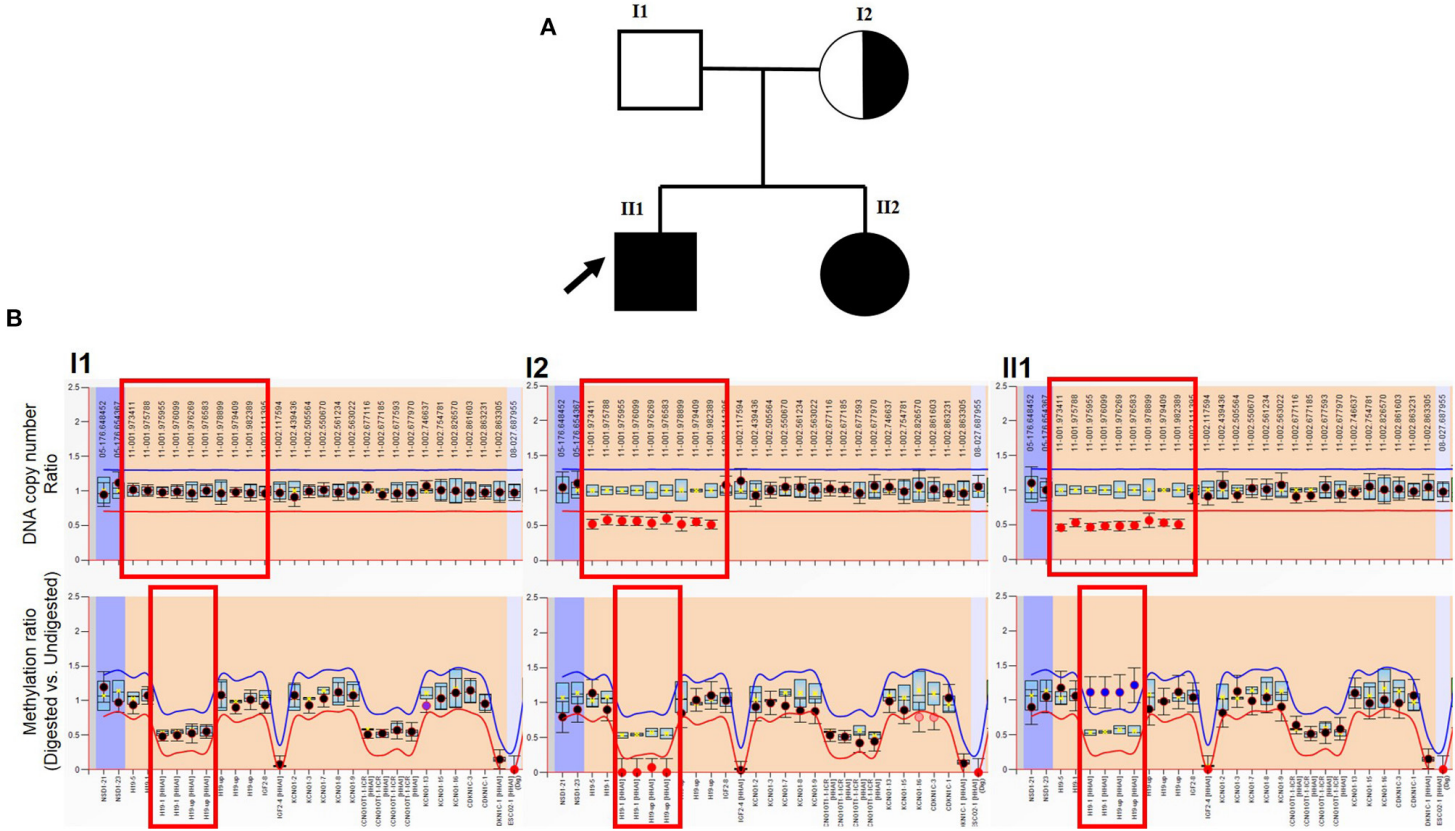
MLPA result of patient. **(A)** Genogram of the BWS family. The arrow points out the male patient (proband) in our study. **(B)** Top row: DNA copy number ratio of patient vs. normal reference. The *X*-axis shows hg18 locations. The red box points out H19 and its upstream location. It shows normal ratio (~1) in the patient's father, and about half (~0.5) in the male patient, and his mother (also in his sister, which is not presented here). Bottom row: methylation ratio of H19. The *X*-axis shows gene exons. The red box points out *H19* and the upstream region. It shows normal ratio (~0.5) in the patient's father. Because his mother lost the methylation copy of *H19*, the methylation level is about 0. The male patient lost the maternal copy of *H19* (also in his sister, which is not presented here), which should not be methylated, which leads to the methylation level increase (~1).

Patient #1 is a 24-year-old man who came to consult about fertility due to orchidopexy in his childhood (Figure 2A). He was diagnosed BWS when he was born based on the features of macroglossia, abdominal wall defects, and bilateral cryptorchidism. He had surgical correction for macroglossia at 6 months of age and orchidopexy at 18 months. Now he is 193 cm tall, and his testis were smaller than usual with volume <8 ml. Multiple-semen analyses showed azoospermia. There were no other abnormalities noted on the annual health examination. Patient #2 is the younger sister of Patient #1, who is 20 years old, diagnosed as BWS as a neonate. She was born with hypoglycemia, macroglossia, and abdominal wall defects and underwent surgical correction when she was 2 years old. Abdominal ultrasound demonstrates a structurally normal uterus and ovaries. There were no other abnormalities noted on the annual health examination. Patients' parents have no physical issues and reported no drugs or abnormal environmental exposures during the pregnancy.

## Materials and Methods

All the patients in this study had been informed and gave their informed consent prior to their inclusion.

DNA was extracted from peripheral blood using QIAamp DNA Blood Mini Kit (Qiagen, USA), following the manufacturer's instructions. All DNA samples were quantified by Qubit™ 1 × dsDNA HS Assay Kit (Thermo Fisher, USA).

To detect the methylation of IC1 and IC2, methylation-specific multiplex ligation-dependent probe amplification (MS-MLPA) was performed using SALSA MS-MLPA Probemix (ME030 BWS/RSS, MRC HOLLAND, Netherlands) following the manufacturer's instructions. The data were analyzed by Coffalyser.NET software (MRC HOLLAND, Netherlands) for MS-MLPA analysis.

To explore whether other genetic mutations might have contributed to cryptorchidism and azoospermia, whole-exome sequencing (WES) (Berry Genomics, China) and detection of Y chromosome microdeletions (Y chromosome microdeletion detection kit, PCR-fluorescence probe method, TOGEN, China) were performed. SNP array (HumanCytoSNP-12 BeadChip, Illumina, USA) was performed to confirm the other aberrant copy number variation in the patient.

## Results

MS-MLPA results showed two abnormal findings: first, the molecular genetic analysis demonstrated a heterozygous deletion of *H19* and its upstream regulatory region [chr11:2016835-2025813 (GRCh37)] in the patients and their mother (Figure 2B). Secondly, the ratio of IC1 methylation was abnormal and associated with a lost copy of the gene. A methylation rate of 50% would be expected in a normal person, whereas it was zero in the mother and 100% in both patients (Figure 2B). These results indicated that the patients' mother likely lost the methylated copy of the IC1 region, and she passed the microdeletion to her children, therefore, the two offspring possess a single methylated copy of IC1 from their father. As the patient and his young sister also demonstrated the loss of the non-methylated copy of IC1, this silenced *H19* gene expression and eventually results in BWS.

The results of Y chromosome microdeletion detection showed no microdeletion in the Y chromosome. WES results showed a heterozygous mutation c.302G>A (p.R101Q) in *PROK2* (prokineticin 2, MIM607002). PROK2 is a newly identified molecular culprit in Kallmann syndrome (KS), and it can be inherited as an autosomal dominant or recessive trait. According to the American College of Medical Genetics and Genomics (ACMG) standards and guidelines, mutation c.302G>A is likely pathogenic. However, this mutation was also found in patients' father, who does not show any symptoms related to KS. Therefore, the association of this mutation with azoospermia is uncertain.

WES and SNP array results were reanalyzed to confirm the breakpoint of the deletion in the patient. The microdeletion appeared to be located around chr11:2009895–2070570 (GRCh37), but the precise breakpoint was not detectable.

## Discussion

Microdeletion and microduplication are uncommon phenomena in BWS patients, accounting for <9% in familial BWS. Sparago et al. (2004) first reported the microdeletion of *H19* DMR in BWS, and several articles demonstrated chromosomal microdeletion in the imprinting center region associated with BWS (Niemitz et al., 2004; Prawitt et al., 2005a,b; Zollino et al., 2010; De Crescenzo et al., 2011; Beygo et al., 2013; Baskin et al., 2014). Two cases in Baskin's study (Baskin et al., 2014) are similar to our patient. One of them is maternal deletion of H19 and IC1 in a patient leading to BWS. The patient is female and also had macrosomia, abdominal wall defects, macroglossia, and neonatal hypoglycemia. However, this girl patient suffered from Wilms tumor, mild nephrosis, nephromegaly, and polydactyly, which were not observed in our patients. The other case in their study is a male patient and had a *de novo* deletion of H19 and IC1. The clinical data is similar to our case except for chryptorchidism and azoospermia, which have been observed in our male patient but not reported in their case. The male patient in our case had chryptorchidism which was corrected by surgery before 2 years old. According to the literature (Feyles et al., 2014), when cryptorchid patients have surgery before age 2, more than 95% patients can reach normal sperm count and motility. However, BWS patients do not appear to experience such high fertility rates with orchidopexy. Gazzin et al. (2019) followed four BWS males who suffered cryptorchidism. All of them had azoospermia after surgery, as in our case. Therefore, azoospermia of our male patient might be not the consequence of cryptorchidism, but due to genetic issues, which lead to dysfunction of the testis.

In recent years, more and more studies focus on the genetic and epigenetic factors in male infertility (Dong et al., 2017; Gunes et al., 2018; Lujan et al., 2019). Some studies showed that abnormal methylation of *H19* may be associated with male infertility. In our case, the male patient lost maternal H19, which thus may be related with his cryptorchidism and azoospermia.

Furthermore, the female patient carried the H19 gene microdeletion, which can be passed on to her children and would lead to BWS. Therefore, it may be reasonable to consider PGT (preimplantation genomic testing) to detect potential for BWS offspring.

In addition, we also reanalyzed WES and SNP array result to find the breakpoint of the DNA deletion for this family. Because of the limitation of these techniques, we need other methods (gap-PCR and Sanger sequencing) to confirm the precise breakpoint of this microdeletion.

## Conclusion

In this study, we report a BWS family, which was due to maternal deletion in *H19* and its upstream regulatory genes. Now that the patients have reached childbearing age, it gives some insight on the presentation of BWS in adulthood and some of the potential reproductive issues. As BWS male patients could face subfertility, these may be associated with specific molecular subtypes. Azoospermia in these patients may not be the consequence of cryptorchidism, but due to genetic or epigenetic issues. Undescended testis greatly increases the risk of several serious complications like testicular torsion and testicular cancer. Therefore, the surgery is probably indicated regardless of its effect on fertility in males with BWS. This report is limited to a small family and thus presents only a portion of possible clinical scenarios. More studies on adult BWS patients are warranted in the future.

## Data Availability Statement

The raw data supporting the conclusions of this article will be made available by the authors, without undue reservation.

## Ethics Statement

The studies involving human participants were reviewed and approved by Reproductive Medical Ethics Committee of Guangzhou Women and Children's medical center. The patients/participants provided their written informed consent to participate in this study.

## Author Contributions

JC designed the study, performed the experiments, analyzed the data, and edited the manuscript. JX and YY collected the clinical information and communicated with patients. LS designed the research, supervised the studies, analyzed the data, and wrote the manuscript. All authors contributed to the article and approved the submitted version.

## Conflict of Interest

The authors declare that the research was conducted in the absence of any commercial or financial relationships that could be construed as a potential conflict of interest.
